# Clinical and laboratory characteristics of children with severe and nonsevere COVID‐19 in Kermanshah, west of Iran: A retrospective study

**DOI:** 10.1002/hsr2.1659

**Published:** 2023-10-31

**Authors:** Fatemeh Nemati Zargaran, Mosayeb Rostamian, Saeed Alimoradi, Shahab Rezaeian, Etrat Javadirad, Roya Chegene Lorestani, Hajar Motamed, Mahtab Hasanpourshahlaei, Elham Rostami, Keyghobad Ghadiri

**Affiliations:** ^1^ Infectious Diseases Research Center, Health Institute Kermanshah University of Medical Sciences Kermanshah Iran; ^2^ Clinical Research Development Center, Taleghani and Imam Ali Hospital Kermanshah University of Medical Sciences Kermanshah Iran; ^3^ Clinical Research Development Center, Imam Khomeini and Mohammad Kermanshahi and Farabi and Imam Reza Hospitals Kermanshah University of Medical Sciences Kermanshah Iran; ^4^ Clinical Research Development Center, Imam Reza Hospital Kermanshah University of Medical Sciences Kermanshah Iran; ^5^ Department Chalous Islamic Azad University Chalous Iran

**Keywords:** children, clinical symptoms, COVID‐19, laboratory findings, pediatrics

## Abstract

**Background and Aims:**

The study aimed to collect and compare clinical and laboratory findings of children with severe and nonsevere COVID‐19 in Kermanshah City, located in the west of Iran.

**Methods:**

The study was conducted on 500 children with COVID‐19 hospitalized in Mohammad‐Kermanshahi Hospital in Kermanshah City. Pediatric COVID‐19 was confirmed by reverse transcription‐polymerase chain reaction (RT‐PCR) test using respiratory secretion samples. Medical records were reviewed and information related to demographic characteristics, underlying diseases, clinical manifestations, laboratory findings, and chest computed tomography (CT) scans were all extracted from electronic and paper records. Patients were divided into three groups according to the severity of the disease: mild, moderate, and severe. Clinical and laboratory findings were compared between the groups and the collected data were analyzed by statistical methods.

**Results:**

Out of 500 patients, 286 were boys and 214 were girls. Of the patients, 321 cases were only COVID‐19, while 179 patients were diagnosed as Multisystem Inflammatory Syndrome in Children (MIS‐C) positive. The average age of COVID‐19 patients was 3.85 ± 4.48 and of MIS‐C patients was 3.1 ± 3.5. In order, fever, cough, and heart disorders were the most common symptoms in patients with COVID‐19 and MIS‐C, respectively. In terms of disease severity, 246 patients had mild disease, 19 patients had moderate disease, and 56 patients had severe disease. In severe patients, the average number of white blood cells (WBC) was higher, while the average number of lymphocytes was lower. Also, in these patients, the average age was lower, and most of them had respiratory distress. In mild patients, often cough, diarrhea, and vomiting were observed.

**Conclusion:**

The results of our study showed that laboratory factors such as WBC count, lymphocyte count, CT findings, Respiratory distress, cough, diarrhea, and vomiting can be used to evaluate the severity of COVID‐19 in children.

## 1 INTRODUCTION

Severe acute respiratory syndrome coronavirus 2 (SARS‐CoV‐2) caused the worldwide COVID‐19 pandemic.^1^ The manifestations of this disease vary from asymptomatic pneumonia to severe respiratory failure and death.^2^ The most common clinical manifestations of COVID‐19 include dry cough, fever, fatigue, shortness of breath, leukopenia, pneumonia, and other respiratory disorders.^3^ Although COVID‐19 affects all ages, according to reports, the spectrum of manifestations in children is different from that of adults,^4^ so the infection in children is usually asymptomatic or there may be a mild infection in the upper respiratory tract, or had some gastrointestinal symptoms, and in severe cases, it may cause coagulation disorders.^5^, ^6^

The prevalence of COVID‐19 in children is reported to be less than 2%,^7^ but the reason for this low prevalence is not clear, whether it is due to its being asymptomatic or due to the reduced susceptibility of children. Hypotheses have been reported in this regard, which include differences in the immune system of children compared with adults, less exposure to the infectious agent, the presence of other viruses in the respiratory tract of children that cause competition between the viruses, and finally the expression of an age‐related angiotensin‐converting enzyme 2 (ACE2).^8^ Although the COVID‐19 prevalence in children is less and they are usually affected in a mild form, if they are infected, they can be carriers for the spread of this dangerous human pathogen.^9^, ^10^

Due to the variety of clinical and laboratory findings, it is difficult to diagnose the COVID‐19 disease in children who have no history of encountering an infected person.^11^ Compared with adults, information on the laboratory findings and clinical characteristics of COVID‐19 is scarce,^12^ so a careful study of the available evidence of characteristics in terms of clinical symptoms, biomarkers, and radiographic images can be helpful. Therefore, the present study aimed to collect and compare clinical and laboratory findings of children with severe and nonsevere COVID‐19 in Kermanshah City, located in the west of Iran.

## 2 METHODOLOGY

This retrospective cross‐sectional study was conducted from August to October 2021 on 500 confirmed cases of children with COVID‐19 hospitalized in Mohammad‐Kermanshahi Hospital in Kermanshah City in the west of Iran. Inclusion criteria included age less than 18 years and a positive result of the COVID‐19 reverse transcription‐polymerase chain reaction (RT‐PCR) test of respiratory secretions obtained through the nasopharyngeal or oropharyngeal swab. Exclusion criteria included incomplete clinical/laboratory data and diagnosis of other pulmonary infections.

Medical records were reviewed and all data related to laboratory findings and other information including demographic characteristics, disease severity, date of onset of symptoms, date of diagnosis, the history of contact with COVID‐19 patients, date of hospitalization, and clinical outcome were extracted. Information on underlying diseases, clinical manifestations (such as cough, fever, and dyspnea), laboratory findings (such as erythrocyte sedimentation rate [ESR], C‐reactive protein [CRP], lymphocyte count, neutrophil count, and oxygen saturation), and chest CT scans were all extracted from electronic medical records.

The patients included in the study were classified into three groups according to the severity of the disease. Severity was defined according to the report provided by the new coronavirus pneumonia prevention and control program (6th edition) published by the National Health Commission of China.^13^ The clinical classification is as follows: (1) Mild symptoms and normal or nonpneumonic findings on radiographic examination, (2) Respiratory symptoms and fever, with evidence of pneumonia on radiographic examination, (3) Shortness of breath, respiratory rate ≥30/min, blood oxygen saturation ≤93%, PaO2/FiO2 ratio 50% of lung field in 24–48 h, and (4) Critical: respiratory failure, requires mechanical ventilation, septic shock, multiple organ dysfunction/failure, and requires ICU monitoring and treatment.

The study was approved by the Institutional Review Board and Ethical Committee of Kermanshah University of Medical Sciences (ethical code: IR.KUMS.REC.1399.681).

### 2.1 Statistical analyses

All analyses were done using SPSS software version 23.0. The normality distribution of the data was checked using the Kolmogorov–Smirnov test. Continuous variables were presented as mean ± standard deviation and analyzed using an independent *t* test/Mann–Whitney *U* test. Categorical variables were presented as counts (percentages) and analyzed with Fisher's test. All tests were two‐sided and *p* < 0.05 was considered statistically significant.

## 3 RESULTS

In total, out of 500 children with suspected COVID‐19 and/or MIS‐C disease hospitalized in Mohammad‐Kermanshahi Hospital, 286 patients (57.2%) were boys and 214 patients (42.8%) were girls. The average age of the patients was 3.85 ± 4.48 years. In terms of age distribution, 146 patients (29.2%) were under 1 year old, 223 patients (44.6%) were between 1 and 5 years old, 103 patients (20.6%) were between 6 and 12 years old, and 28 patients (5.6%) were 12 years old and older.

Out of 500 patients, 321 (64.2%) were COVID‐19 patients and 179 (35.8%) were MIS‐C cases. Clinical manifestations and laboratory findings in patients with COVID‐19 and MIS‐C are presented in Tables 1 and 2, respectively. The most common symptoms were fever in 148 (29.6%) cases, followed by dry cough in 139 cases (27.8%). Gastrointestinal disorders were also observed in patients, such that vomiting was observed in 71 patients (14.2%) and diarrhea in 88 patients (17.6%). Neurological disorders including loss of consciousness in 5 patients (1%), seizures in 15 patients (3%), and headache in 21 patients (4.2%) were observed. Ten patients (11%) showed skin lesions.

**Table 1:** The laboratory findings of the patients with COVID‐19 and MIS‐C at admission time.

Variable	COVID‐19	MIS‐C
	Mean ± SD	
Age (years)	3.85 ± 4.48	3.1 ± 3.5
Weight (kg)	17.46 ± 16.46	16.7 ± 13.2
WBC (10^3^/µL)	10.16 ± 7.71	13.87 ± 46.12
Neutrophil (%)	52.74 ± 21.06	52.9 ± 17
Lymphocytes (%)	42.71 ± 20.76	42.4 ± 16.9
ESR (33/h)	26.43 ± 22.01	26.28 ± 22.64
D‐dimer (ng/mL)	855.38 ± 1187.66	1161.9 ± 2262.2
ALT (U/L)	37.28 ± 65.51	36.9 ± 42.5
AST (U/L)	47.71 ± 84.93	40.4 ± 19.4
RR	30.03 ± 12.91	25.85 ± 5.86
Duration‐hospitalization (day)	3.92 ± 3.49	4 ± 3.4
Symptom‐duration (day)	3.06 ± 2.69	3.4 ± 2.4
Temperature (°C)	37.38 ± 0.48	37.3 ± 2.8

**Table 2:** Clinical symptoms, diagnosis, and treatment of the patients with COVID‐19 and MIS‐C.

	Total		COVID‐19		MIS‐C	
	Positive	Negative	Positive	Negative	Positive	Negative
Chest pain	6 (1.2)	494 (98.8)	3 (0.9)	318 (99.1)	3 (1.7)	176 (98.3)
Respiratory distress	83 (16.6)	417 (83.4)	72 (22.4)	249 (77.6)	11 (6.2)	168 (93.8)
Intubation	11 (2.2)	489 (97.8)	10 (3.1)	311 (96.9)	1 (0.5)	178 (99.5)
Oxygen Therapy	44 (8.8)	456 (91.2)	38 (11.8)	283 (88.2)	6 (3.5)	173 (96.5)
Heart condition	189 (37.8)	311 (62.2)	84 (26.2)	237 (73.8)	105 (58.6)	74 (41.4)
Muscle pain	21 (4.2)	479 (95.8)	12 (3.7)	309 (96.3)	9 (5.1)	170 (94.9)
Underlying‐disease	69 (13.8)	431 (86.2)	54 (16.8)	267 (83.2)	15 (8.4)	164 (91.6)
Cough	139 (27.8)	361 (72.2)	95 (29.6)	226 (70.4)	44 (24.6)	135 (75.4)
Loss of consciousness	5 (1)	495 (99)	2 (0.6)	319 (99.4)	3 (1.7)	176 (98.3)
Convulsion	15 (3)	485 (97)	12 (3.7)	309 (96.3)	3 (1.7)	176 (98.3)
bellyache	38 (7.6)	462 (92.4)	26 (8.1)	295 (91.9)	12 (6.7)	167 (93.3)
Nausea	31 (6.2)	469 (93.8)	24 (7.5)	297 (92.5)	7 (3.9)	172 (96.1)
Vomiting	71 (14.2)	429 (85.8)	47 (14.6)	274 (85.4)	24 (13.4)	155 (86.6)
Diarrhea	88 (17.6)	412 (82.4)	56 (17.4)	265 (82.6)	32 (17.9)	147 (82.1)
Anorexia	55 (11)	445 (89)	35 (10.9)	286 (89.1)	20 (11.2)	159 (88.8)
Headache	21 (4.2)	479 (95.8)	12 (3.7)	309 (96.3)	9 (5.1)	170 (94.9)
Hangover	6 (1.2)	494 (98.8)	2 (14.6)	319 (99.4)	2 (1.1)	177 (98.9)
Plasy‐organs	1 (0.2)	499 (99.8)	1 (0.3)	320 (99.7)	0 (0)	179 (100)
Skin_lesion	10 (11)	490 (89)	5 (1.5)	316 (98.5)	5 (2.8)	174 (97.2)
Cancer	6 (1.2)	494 (98.8)	6 (1.8)	315 (98.2)	0 (0)	179 (100)
Chronic liver	1 (0.2)	499 (99.8)	1 (0.3)	320 (99.7)	0 (0)	179 (100)
Diabetes	3 (0.6)	497 (99.4)	3 (0.9)	318 (99.1)	0 (0)	179 (100)
Chronic blood	1 (0.2)	499 (99.8)	1 (0.3)	320 (99.7)	0 (0)	179 (100)
Chronic disease	22 (4.4)	488 (97.6)	22 (6.8)	299 (93.2)	0 (0)	179 (100)
Death	8 (1.6)	492 (98.4)	8 (2.5)	313 (97.5)	0 (0)	179 (100)
Contact history	132 (26.4)	368 (73.6)	81 (25.2)	240 (74.8)	51 (28.5)	128 (71.5)
CRP	246 (49.2)	254 (50.8)	169 (52.6)	152 (47.4)	77 (43.1)	102 (56.9)
ASA	168 (33.6)	332 (66.4)	58 (10.1)	263 (81.9)	110 (61.4)	69 (38.6)
IVIG	71 (14.2)	429 (85.8)	29 (9.1)	292 (90.9)	42 (23.5)	137 (76.5)
Prednisolone	190 (38)	310 (62)	67 (20.9)	254 (79.1)	123 (23.5)	56 (76.5)
Enoxaparin	30 (6)	470 (94)	16 (4.9)	305 (95.1)	14 (7.8)	165 (92.2)
Pantoprazole	141 (28.2)	359 (71.8)	79 (24.6)	242 (75.4)	62 (34.6)	117 (65.4)
Remdesivir	24 (4.8)	476 (95.2)	20 (6.2)	301 (93.8)	4 (2.2)	175 (97.8)

In terms of laboratory factors, the average white blood cells (WBC) and D‐dimer were higher in patients with MIS‐C compared with COVID‐19 patients.

The COVID‐19 patients (321 cases) were classified according to the severity of the disease as follows: 246 cases (76.6%) were mild, 19 cases (5.9%) were moderate, and 56 cases (17.4%) were severe. Critical diseases was not observed in any of the pediatrics. The comparison of clinical and laboratory characteristics based on the severity of the disease is presented in Tables 3 and 4.

**Table 3:** Comparing the clinical symptoms, laboratory parameters, and treatment of pediatric with COVID‐19 according to the severity of the disease.

	Total	Mild	Moderate	Severe	*p* Value
Chest‐pain					
Yes	3 (0.9)	0 (0)	2 (10.5)	1 (1.8)	0.001
No	318 (99.1)	246 (100)	17 (89.4)	55 (98.2)	
Po2					
<93	56 (17.4)	8 (3.25)	6 (31.5)	42 (75)	0.001
>93	265 (82.6)	238 (96.7)	13 (68.4)	14 (25)	
Respiratory‐distress					
Yes	72 (22.4)	16 (6.5)	10 (52.6)	46 (82.1)	0.001
No	249 (77.6)	230 (93.5)	9 (47.3)	10 (17.8)	
Intubation					
Yes	10 (3.1)	4 (1.6)	0 (0)	6 (10.7)	0.001
No	311 (96.9)	242 (98.3)	19 (100)	50 (89.2)	
Oxygen‐therapy					
Yes	38 (11.8)	2 (0.8)	8 (42.1)	28 (50)	0.001
No	283 (88.2)	244 (99.2)	11 (57.8)	28 (50)	
CTX					
Annormal	67 (20.9)	1 (0.4)	15 (78.9)	51 (91)	0.001
Normal	43 (13.4)	42 (17)	0 (0)	1 (1.7)	
Not done	211 (65.7)	203 (82.5)	4 (21)	4 (7.1)	
Heart‐condition					
Yes	84 (26.2)	54 (22)	5 (26.3)	25 (44.6)	0.001
No	237 (73.8)	192 (78)	14 (73.6)	31 (55.3)	
Muscle‐pain					
Yes	12 (3.7)	9 (3.6)	0 (0)	3 (5.4)	0.5
No	309 (96.3)	237 (96.3)	19 (100)	53 (94.6)	
Underlying‐disease					
Yes	54 (16.8)	38 (15.4)	2 (10.5)	14 (25)	0.1
No	267 (83.2)	208 (84.5)	17 (89.4)	42 (75)	
Cough					
Yes	95 (29.6)	71 (28.9)	12 (63.1)	12 (21.4)	0.001
No	226 (70.4)	175 (71.1)	7 (36.8)	44 (78.6)	
Decrease wareness level					
Yes	2 (0.6)	2 (0.8)	0 (0)	0 (0)	0.7
No	319 (99.4)	244 (99.2)	19 (100)	56 (100)	
Convulsion					
Yes	12 (3.7)	9 (3.7)	0 (0)	3 (5.4)	0.5
No	309 (96.3)	237 (96.3)	19 (100)	53 (94.6)	
Bellyache					
Yes	26 (8.1)	23 (9.3)	1 (5.2)	2 (3.6)	0.3
No	295 (91.9)	223 (90.7)	18 (94.7)	54 (96.4)	
Nausea					
Yes	24 (7.5)	20 (8.2)	0 (0)	4 (7.1)	
No	297 (92.5)	225 (91.8)	19 (100)	53 (92.9)	
Vomiting					
Yes	47 (14.6)	43 (17.5)	1 (5.2)	3 (5.4)	0.01
No	274 (85.4)	203 (82.5)	18 (94.7)	53 (94.6)	
Diarrhea					
Yes	56 (17.4)	51 (20.7)	3 (15.7)	2 (3.6)	0.01
No	265 (82.6)	195 (79.3)	16 (84.2)	54 (96.4)	
Anorexia					
Yes	35 (10.9)	33 (13.4)	1 (5.2)	1 (1.8)	0.01
No	286 (89.1)	213 (86.6)	18 (94.7)	55 (98.2)	
Headache					
Yes	12 (3.7)	10 (4.1)	1 (5.2)	1 (1.8)	0.7
No	309 (96.3)	236 (95.9)	18 (94.7)	55 (98.2)	
Hangover					
Yes	2 (0.6)	2 (0.8)	0 (0)	0 (0)	0.7
No	319 (99.4)	244 (99.2)	19 (100)	56 (100)	
Plasy‐organ					
Yes	1 (0.3)	1 (0.4)	0 (0)	0 (0)	0.8
No	320 (99.7)	245 (99.6)	19 (100)	56 (100)	
Skin‐lesion					
Yes	5 (1.5)	5 (2)	0 (0)	0 (0)	0.4
No	316 (98.5)	241 (98)	19 (100)	56 (100)	
Cancer					
Yes	6 (1.9)	5 (2)	1 (5.2)	0 (0)	0.3
No	315 (98.1)	241 (98)	18 (94.7)	56 (100)	
Chronic‐liver					
Yes	1 (0.3)	1 (0.4)	0 (0)	0 (0)	0.8
No	320 (99.7)	245 (99.6)	19 (100)	56 (100)	
Diabetes					
Yes	3 (0.9)	3 (1.2)	0 (0)	0 (0)	0.6
No	318 (99.1)	243 (98.8)	19 (100)	56 (100)	
Chronic‐blood					
Yes	1 (0.3)	1 (0.4)	0 (0)	0 (0)	0.8
No	320 (99.7)	245 (99.6)	19 (100)	56 (100)	
Chronic‐disease					
Yes	22 (6.8)	11 (4.5)	1 (5.2)	10 (17.8)	0.001
No	299 (93.2)	235 (95.5)	18 (94.7)	46 (82.2)	
Death					
Yes	8 (2.5)	3 (1.2)	0 (0)	5 (8.9)	0.6
No	313 (97.5)	243 (98.8)	19 (100)	51 (91.1)	
ASA					
Yes	58 (18.1)	41 (16.6)	6 (31.5)	11 (19.6)	0.2
No	263 (81.9)	205 (83.3)	13 (68.4)	45 (68.4)	
IVIG					
Yes	29 (9.1)	18 (7.3)	3 (15.7)	8 (14.2)	0.1
No	292 (90.9)	228 (92.6)	16 (84.2)	48 (85.7)	
CRP (Qualitative)					
Positive	151 (47.1)	121 (49.2)	10 (52.63)	20 (28.67)	0.2
Negative	170 (52.9)	125 (50.8)	9 (47.37)	36 (64.29)	
Contact					
Yes	81 (25.2)	57 (23.1)	10 (52.6)	14 (25)	0.01
No	240 (74.8)	189 (76.8)	9 (47.3)	42 (75)	

**Table 4:** Comparing the demographic data and laboratory parameters according to the severity of the disease.

	Mild	Moderate	Severe	*p* Value
Age (year), mean ± SD	4 ± 4.35	6.3 ± 5.2	2.3 ± 4.3	0.001
RR, mean ± SD	26.2 ± 7.3	26.8 ± 11.1	47 ± 17.6	0.001
Duration hospitalization, mean ± SD (day)	3.4 ± 3	5.5 ± 4.5	5.3 ± 4.2	0.001
Symptom duration, mean ± SD (day)	3.2 ± 2.8	3.2 ± 2.8	2.1 ± 1.8	0.01
Temperature, mean ± SD (°C)	37.4 ± 0.4	37.4 ± 0.5	37.2 ± 0.5	0.1
Weight(kg), mean ± SD	17.5 ± 14.4	30.5 ± 26.3	35.9 ± 174.4	0.2
ALT (U/L), mean ± SD	38.2 ± 73.3	36 ± 20	33.6 ± 33.7	0.1
AST (U/L), mean ± SD	48.2 ± 94.9	42.4 ± 14.3	47.3 ± 46.4	0.1
PCO2, mean ± SD	31.7 ± 12.6	30.4 ± 7.1	34.3 ± 10.4	0.5
PR, mean ± SD	117.3 ± 16.6	119.3 ± 19	135.2 ± 19.1	0.001
BP, mean ± SD	91.7 ± 12.4	92.4 ± 11.5	92.1 ± 18.1	0.1
WBC (10^3^/µL), mean ± SD	9.5 ± 4.6	15.5 ± 24.2	11.2 ± 7	0.001
Neutrophil(%), mean ± SD	51.4 ± 21.2	62.6 ± 19.5	55 ± 20.1	0.06
Lymphocytes(%), mean ± SD	44.2 ± 20.7	33 ± 19.4	39.7 ± 20.6	0.04
ESR(mm/h), mean ± SD	26.9 ± 22.6	28.4 ± 13.6	19.9 ± 20.1	0.3
D‐dimer(ng/mL), mean ± SD	862.1 ± 1329.3	831.6 ± 929	848.2 ± 737	0.99

A significant difference was observed between the severe and mild groups in terms of age, The average age children in the severe group (2.3 ± 4.3) were significantly longer had a lower than in mild patients (4 ± 4.35) (*p* = 0.001).

The mean hospitalization days in severe patients (5.3 ± 4.2 days) were significantly longer than in mild patients (3.4 ± 3 days) (*p* = 0.001).

A significant difference was observed between the severe and mild groups in terms of clinical symptoms such as chest pain, po2, respiratory distress, incubation requirement, oxygen therapy, CT scan, heart disorder, cough, vomiting, diarrhea, anorexia, and underlying disease (Table 3). In addition, in terms of laboratory factors, a significant difference was observed between the severe and mild groups in terms of the number of WBC and the number of lymphocytes (*p* = 0.003 and *p* = 0.04, respectively) (Table 4).

The findings obtained from the chest CT scan according to the severity of the disease are presented in Table 3. The severe group had more abnormal CT findings (51 cases, 91%) than the mild group (one case, 0.4%) and moderate group (15 cases, 78.9%) (*p* = 0.001).

In patients with MIS‐C, a chest X‐ray was performed on 36 patients, 15 of whom had abnormal symptoms and 21 had no symptoms.

Antiviral anticoagulant drugs used in patients included aspirin (ASA) in 168 patients (33.6%), IVIG in 71 patients (14.2%), prednisolone in 190 patients (38%), enoxaparin in 30 patients (6%), and remdesivir in 24 patients (4.8%).

The sensitivity and specificity indices for a number of variables to predict the severity of the disease are shown in Table 5.

**Table 5:** Sensitivity and specificity indicators for the speculation of disease severity.

	Age	Lymphocytes	PR	WBC	Duration of hospitalization	RR	Symptom duration
*Moderate*							
Optimal cut point	5.5	47.5	127	11.13	4.5	27.5	2.5
Sensitivity	0.53	0.33	0.37	0.39	0.63	0.37	0.42
Specificity	0.71	0.54	0.77	0.68	0.74	0.72	0.49
AUC	0.62	0.44	0.57	0.54	0.68	0.55	0.46
*Severe*							
Optimal cutpoint	7.5	49.5	127	9.45	4.5	33.5	2.5
Sensitivity	0.2	0.41	0.73	0.5	0.52	0.78	0.3
Specificity	0.8	0.57	0.77	0.57	0.74	0.9	0.49
AUC	0.5	0.49	0.75	0.53	0.63	0.84	0.4

## 4 DISCUSSION

Considering the limited information on the clinical characteristics and laboratory findings of COVID‐19 in children, here we conducted a retrospective study of COVID‐19 in people less than 18 years of age in Kermanshah City. In our study, most children (44.6%) were in the age group of 1–5 years. Similarly, in the Martinez‐Garcia study, the 1–5‐year‐old group had the highest prevalence of COVID‐19 compared with other children.^14^ In the study of Sharifi et al., 52% of the children were under 5 years of age.^15^ In Dong et al.'s study, 40% of children were under 5 years of age.^16^ The high prevalence of infection in this age group compared with other children is due to the problems in infection control, including their less use of masks and their high contact with adults due to their need for care.

Previous studies showed that the clinical symptoms in infants under 1 year were more severe than in other children's age groups.^17^ In the present study, there was a statistically significant relationship between the severity of the infection and age, so that the average age of the patients was lower in the group where the infection was more severe. In the study of Najafinejad et al., it was also reported that most of the confirmed and severe cases occurred in infants under 1 year of age.^18^

The findings of our study showed that boys are slightly more affected by COVID‐19 disease, so that the COVID‐19 incidence rate in boys was 57.2% and in girls was 42.8%. Similar to our study, other studies reported more COVID‐19 cases in boys.^16^, ^19^, ^20^, ^21^, ^22^ This difference may be due to genetic, immunological, and hormonal differences.^23^

A history of contact with family members who were infected with COVID‐19 or going to an epidemic area are among the causes of the disease spread in children.^16^, ^21^ In our study, only 26.4% mentioned contact history. Parri et al. observed that 53.8% of positive cases had previous contact with a COVID‐19 case.^24^ In the studies by Goktug et al.^11^ and Hoang et al.,^25^ 49.6% and 75.6% of children had a confirmed history of contact with a COVID‐19 case, respectively. In the present study, most of the people did not mention contact history, which is because most of them are young and cannot express their conditions well, or because of the wrong information given by their parents. The difference between the studies in this regard may be due to conducting the study in different epidemic times or the difference in the measures taken by the government and families.

In children, the clinical course of COVID‐19 is mild and has a wide clinical spectrum.^3^, ^25^, ^26^ In our study, cough, diarrhea, vomiting, and respiratory distress were the most common symptoms, followed by skin lesions, abdominal pain, muscle pain, headache, and seizures. In previous reports, the most common symptoms were fever and cough.^12^, ^16^, ^18^, ^21^, ^22^, ^27^ According to the results of a systematic review by De Souza et al., fever was the most common symptom, followed by cough, diarrhea, nausea, vomiting, fatigue, and respiratory distress.^28^ Gastrointestinal complaints such as nausea and vomiting, abdominal pain, and diarrhea have also been reported in some studies.^18^, ^29^, ^30^, ^31^ There are also reports of skin lesions in pediatric COVID‐19 patients.^18^, ^29^, ^32^, ^33^, ^34^ In addition, one of the main symptoms of pediatric COVID‐19 is myalgia.^35^, ^36^, ^37^ Nervous symptoms including headache, dizziness, and convulsions, have been also reported in some studies.^29^, ^38^

In our study, decreased sense of smell and taste was not seen in any of the patients. These symptoms are usually less investigated in children. In Goktug et al.'s study, there was a loss of smell in 3.5% and a loss of taste in 4.3%, and all patients were over 10 years old.^11^ In a study in Turkey, loss of smell/taste was seen in 5.7% of cases.^39^ In our study, 73.8% of patients were <5 years old, and were unable to describe their sense of smell and taste, accurately.

In the severe group, higher chest pain, respiratory distress, breathing rate, pulse rate, underlying disease, cardiac involvement, oxygen therapy, longer hospitalization time, and lower oxygen saturation percentage were observed. In the mild group, cough, diarrhea, and vomiting were more prevalent. Similar to our study, the results of Hosseini Yazdi et al.'s study also showed that patients in the severe group had longer hospital stays and lower oxygen saturation than nonsevere respiratory distress patients.^40^

In the present study, the inflammatory markers of CRP and ESR were not predictive factors for the severity of the COVID‐19 disease. In the study of Najafinejad et al., like our study, half of the people with COVID‐19 had positive CRP, but no correlation was observed between CRP and the severity of the disease.^18^ On the contrary, the number of WBC and lymphocytes was related to the severity of the disease of COVID‐19. Most previous studies have also shown a decrease in lymphocytes in patients with severe infection.^41^, ^42^, ^43^ DeBiasi et al. also expressed lymphopenia as a factor to determine the severity of pediatric COVID‐19.^44^ It should be mentioned that the difference in laboratory parameters can be due to differences in the severity of the infection of the patients and the time from the onset of symptoms to the completion of the test.

Studies reported that COVID‐19 is more common in younger children and children with respiratory dysfunction. However, patients with MIS‐C who often present with cardiac dysfunction and gastrointestinal symptoms do not have a specific underlying disease.^45^, ^46^ Inflammatory markers increase significantly in patients with MIS‐C.^47^ In our study, most of the children with MIS‐C had cardiac dysfunction and some of them also had digestive disorders. Also, regarding inflammatory markers, in 52% of COVID‐19 patients and 43% of MIS‐C patients CRP was positive. The average ESR was also high in COVID‐19 and MIS‐C patients. The mean WBC count (10^3^/µL) was 13.8 and 10.16 in MIS‐C and COVID‐19 patients, respectively. Compared with COVID‐19 patients, the amount of D‐dimer was higher in MIS‐C patients. In the study by Yasuhara et al., patients with MIS‐C usually had cardiac involvement, elevated inflammatory markers, gastrointestinal symptoms, and skin manifestations.^1^

Antiviral treatments and supportive care are essential for patients with COVID‐19.^13^ In the present study, IVIG was prescribed in 9.1% of patients with COVID‐19 and 23.5% of patients with MIS‐C. Also, ASA was used in 10.1% and 61.4% of patients with COVID‐19 and MIS‐C, respectively. Enoxaparin was also used in 6% of patients. Shahbaz‐Nejad et al. reported that 13 cases of children with MIS‐C were prescribed IVIG.^48^ This drug reduces the risk of coronary artery disease.^49^ The decision to treat with anticoagulant drugs in children with COVID‐19 and MIS‐C can prevent the adverse consequences of the diseases.^50^

In our study, the death rate was eight cases (1.6%). Using the data of the national COVID‐19 registry for all positive COVID‐19 cases in Iran, Madani et al. reported a 5.3% mortality rate in people under the age of 18 from January 2020 to October 2020.^51^ In a cross‐sectional study, Shahbaz‐Nejad et al. reported a mortality rate of 4% of 100 COVID‐19 children admitted to hospitals in Gilan province (north of Iran) from February 02, 2020 to June 28, 2020.^48^ In Armin et al.'s study, which was conducted from March 20, 2020 to March 20, 2021, in the cities of Tehran, Isfahan, Ahvaz, Bandar Abbas, and Khorram Abad, the mortality rate of 278 children infected with COVID‐19 was 4.3%.^19^ In a study conducted from March 10, 2020 to June 28, 2020, by Foster et al. in the United States, examining 1215 patients under 21 years of age with COVID‐19, two patients (0.16%) died.^52^ In Zheng's study, none of the patients died due to COVID‐19.^25^ Yayla et al. reported a mortality rate of 1.3% from March 11, 2020 to March 23, 2020, examining 77 cases of children with COVID‐19 admitted to a hospital in Ankara.^39^ These differences in the percentage of mortality in different studies can be due to the difference in the age of the patients, underlying diseases, different times of the epidemic, and the severity of the disease.

Among the limitations of this study, the following can be mentioned: (1) This study only included a short 3‐month observational design with a retrospective nature, (2) The information was based on patient records and there was some missing data, (3) CT scan and imaging was only performed in a few patients and these results cannot reflect the results of all patients, (4) Not all the laboratory tests were performed in all patients.

## 5 CONCLUSIONS

The results of our study showed that laboratory factors such as WBC count, lymphocyte count, CT findings, Respiratory distress, cough, diarrhea, and vomiting can be used to evaluate the severity of COVID‐19 in children.

## AUTHOR CONTRIBUTIONS

**Fatemeh Nemati Zargaran**: Data curation; writing—original draft. **Mosayeb Rostamian**: Writing—original draft. **Saeed Alimoradi**: Data curation. **Shahab Rezaeian**: Formal analysis; software. **Etrat Javadirad**: Writing—review and editing. **Roya Chegene Lorestani**: Data curation; writing—original draft; writing—review and editing. **Hajar Motamed**: Writing—review and editing. **Mahtab Hasanpourshahlaei**: Data curation. **Elham Rostami**: Data curation. **Keyghobad Ghadiri**: Conceptualization; Writing—review and editing.

## CONFLICT OF INTEREST STATEMENT

The authors declare no conflict of interest.

## TRANSPARENCY STATEMENT

The lead author Keyghobad Ghadiri affirms that this manuscript is an honest, accurate, and transparent account of the study being reported; that no important aspects of the study have been omitted; and that any discrepancies from the study as planned (and, if relevant, registered) have been explained.

## Supporting information

Supporting information.

## Data Availability

All data are relevant to the study are included in the article or uploaded as supplementary information. The data sets used and/or analyzed during the current study are available from the corresponding author upon reasonable request
